# The Leu/Val^6.51^ Side Chain of Cannabinoid Receptors Regulates the Binding Mode of the Alkyl Chain of Δ^9^-Tetrahydrocannabinol

**DOI:** 10.1021/acs.jcim.3c01054

**Published:** 2023-08-30

**Authors:** Claudia Llinas del Torrent, Iu Raïch, Angel Gonzalez, Nil Casajuana-Martin, Jaume Lillo, Joan Biel Rebassa, Carlos Ferreiro-Vera, Verónica Sánchez de Medina, Rafael Franco, Gemma Navarro, Leonardo Pardo

**Affiliations:** † Laboratory of Computational Medicine, Biostatistics Unit, Faculty of Medicine, Universitat Autònoma Barcelona, 08193 Bellaterra, Barcelona, Spain; ‡ Department of Biochemistry and Molecular Biomedicine, School of Biology, University of Barcelona, 08028 Barcelona, Spain; § Centro de Investigación en Red, Enfermedades Neurodegenerativas (CIBERNED), Instituto de Salud Carlos III, 28031 Madrid, Spain; ∥ Department of Biochemistry and Physiology, Faculty of Pharmacy and Food Sciences, Universitat de Barcelona, 08028 Barcelona, Spain; ⊥ Phytoplant Research S.L.U., 14014 Córdoba, Spain; # Institute of Neuroscience, University of Barcelona (NeuroUB), Av Joan XXIII 27-31, 08028 Barcelona, Spain

## Abstract

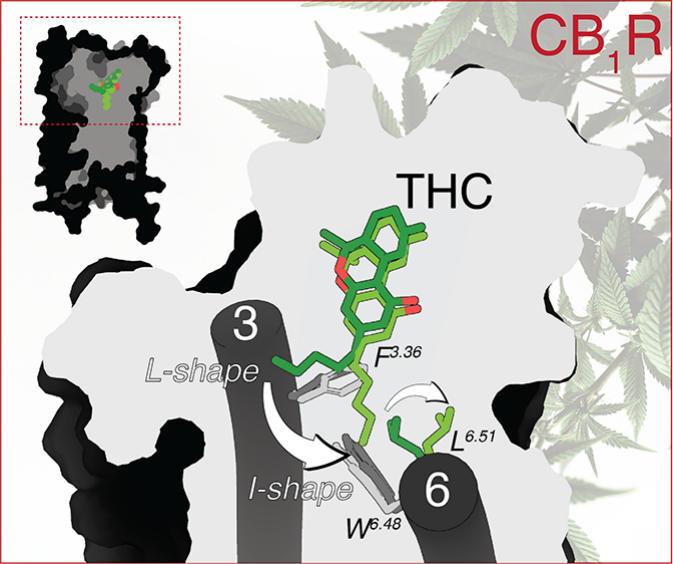

(−)-Δ^9^-*trans*-tetrahydrocannabinol (THC), which is the principal psychoactive constituent of *Cannabis*, mediates its action by binding to two members of the G-protein-coupled receptor (GPCR) family: the cannabinoid CB_1_ (CB_1_R) and CB_2_ (CB_2_R) receptors. Molecular dynamics simulations showed that the pentyl chain of THC could adopts an I-shape conformation, filling an intracellular cavity between Phe^3.36^ and Trp^6.48^ for initial agonist-induced receptor activation, in CB_1_R but not in CB_2_R. This cavity opens to the five-carbon chain of THC by the conformational change of the γ-branched, flexible, Leu^6.51^ side chain of CB_1_R, which is not feasible by the β-branched, mode rigid, Val^6.51^ side chain of CB_2_R. In agreement with our computational results, THC could not decrease the forskolin-induced cAMP levels in cells expressing mutant CB_1_R^L6.51V^ receptor but could activate the mutant CB_2_R^V6.51L^ receptor as efficiently as wild-type CB_1_R. Additionally, JWH-133, a full CB_2_R agonist, contains a branched dimethyl moiety in the ligand chain that bridges Phe^3.36^ and Val^6.51^ for receptor activation. In this case, the substitution of Val^6.51^ to Leu in CB_2_R makes JWH-133 unable to activate CB_2_R^V6.51L^. In conclusion, our combined computational and experimental results have shown that the amino acid at position 6.51 is a key additional player in the initial mechanism of activation of GPCRs that recognize signaling molecules derived from lipid species.

## 1 Introduction

**Figure 1:**
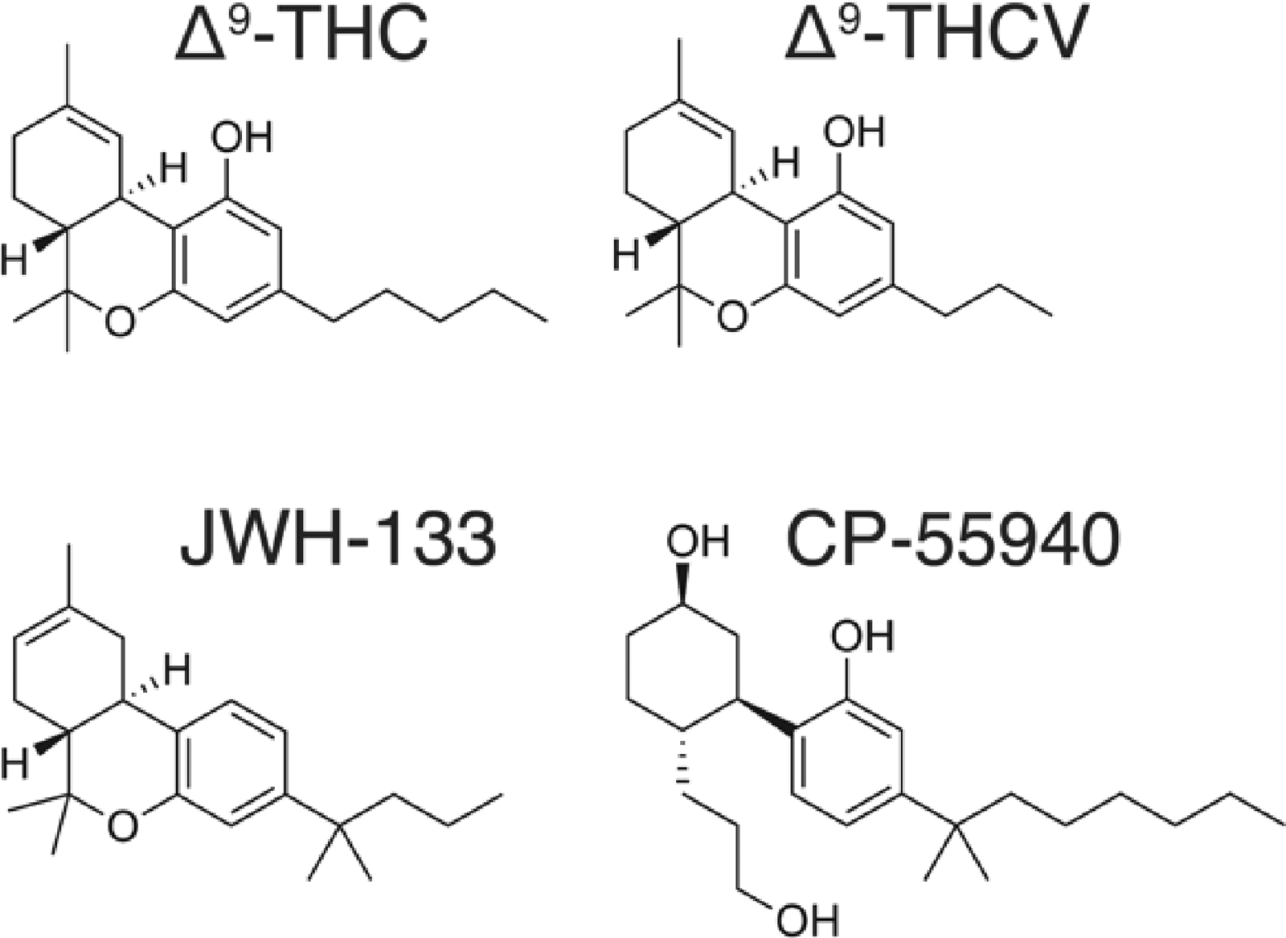
Structures of Δ^9^-THC, Δ^9^-THCV, JWH-133, and CP-55940.

Cannabinoids are naturally occurring compounds found in the *Cannabis sativa* plant (more commonly known as marijuana). There are over 180 cannabinoids out of the 1600 chemical compounds that have been isolated from *Cannabis*, with a characteristic oxygen containing C_21_ aromatic hydrocarbons.^1^ These exogenous cannabinoids can be further classified into 11 subclasses: cannabichromene (CBC), cannabidiol (CBD), cannabielsoin (CBE), cannabigerol (CBG), cannabicyclol (CBL), cannabinol (CBN), cannabinodiol (CBND), cannabitriol (CBT), (−)-Δ^8^-trans-tetrahydrocannabinol (Δ^8^-THC), (−)-Δ^9^-trans-tetrahydrocannabinol (Δ^9^-THC), and miscellaneous-type cannabinoids.^2^ The Δ^9^-THC subclass contains 25 compounds with common structural features such as a dibenzopyran ring and a hydrophobic alkyl chain. This class includes the most abundant phytocannabinoids: (−)-Δ^9^-*trans*-tetrahydrocannabinol (THC), which is the principal psychoactive constituent of *Cannabis*, and (−)-Δ^9^-trans-tetrahydrocannabivarin (THCV), which is homologous to THC but has a 3-carbon (propyl chain) instead of a 5-carbon (pentyl chain) in the alkyl chain (Figure 1). THCV lacks the psychoactive effects of THC and upregulates energy metabolism, converting it a clinically useful remedy for weight loss, obesity management, and type 2 diabetic patients.^3,4^ In addition, THCV can produce beneficial antipsychotic effects.^5^ Endogenous cannabinoids are *N*-arachidonylethanolamide (anandamide) and 2-arachidonoylglycerol (2-AG) that possess long hydrophobic moieties.^6^

The effects of cannabinoids are primarily mediated through two members of the G-protein-coupled receptor (GPCR) family,^7^ the cannabinoid CB_1_ (CB_1_R) and CB_2_ (CB_2_R) receptors. CB_1_R is one of the most abundant GPCRs in the central nervous system, whereas CB_2_R is mainly expressed in the immune system.^8^ However, other molecular targets for certain cannabinoids, aside from CB_1_R and CB_2_R, have also been identified.^9^ Some authors propose that THCV is a CB_1_R and CB_2_R antagonist,^10^ whereas others suggest that THC and THCV are partial agonists at both receptors.^11^ We have recently shown that THC acts as a partial agonist in CB_1_R and as an antagonist in CB_2_R, whereas THCV acts as an antagonist on both receptors.^12^

Here, we have used the recently released structures of CB_1_R^13−15^ and CB_2_R^16−18^ in their inactive and active, G_i_-bound, conformations to delineate the individual signaling contributions of THC and THCV to modulate both receptors. Phe^3.36^ and Trp^6.48^ have been described as conformational toggle or trigger switches involved in the initial agonist-induced receptor activation in CB_1_R,^15,19^ CB_2_R^16^ and other GPCRs.^20−22^ However, these amino acids are conserved in CB_1_R and CB_2_R (44% sequence identity between receptors), thus they cannot explain the different pharmacological profile of THC and THCV. In this manuscript, a combination of molecular dynamics (MD) simulations and site-directed mutagenesis have permitted to propose residue at position 6.51, which is Leu at CB_1_R and Val at CB_2_R, as an additional player capable to selectively recognize the alkyl chain of these ligands, further supporting the yin-yang functional relationship already described for CB_1_ and CB_2_ receptors.^16^ This knowledge could be of great use to facilitate the future design of selective drugs in the endocannabinoid system.

## 2 Materials and Methods

### 2.1 Initial CB_1_R and CB_2_R Models

The CB_1_R-AM841-G_i_ (PDB id 6KPG) and CB_2_R-AM12033-G_i_ (6KPF) cryo-EM structures^18^ were used in docking studies and MD simulations. Missing residues 55–180 of α_i_ in the CB_1_R-AM841-G_i_ structure were built from the structure of G_i_ (6CRK);^23^ and missing residues 55–181 and 233–239 of α_i_ in the CB_2_R-AM12033-G_i_ structure were built from the CB_2_R-WIN55,212–2-G_i_ structure (6PT0),^17^ using AutoModel class^24^ of MODELER v10.1.^25^ Protonation states were assigned with the PDB 2PQR tool^26^ using PROPKA to predict the p*K*_a_ values of ionizable groups in the proteins at pH 6.5.^27^ Disulfide bonds between cysteines were built using the tleap module of Ambertools19. Internal water molecules were added to CB_1_R and CB_2_R using HomolWat.^28^ THC and THCV were docked into the orthosteric binding cavity of CB_1_R and CB_2_R and JWH-133 into CB_2_R by using AM841 in 6KPG and AM12033 in 6KPF structures as a reference. Thus, the alkyl chains of THC, THCV, and JWH-133 were initially modeled in the L-shape conformation above Trp279^5.43^ and Trp194^5.43^ of CB_1_R and CB_2_R, respectively, as observed in the cryo-EM structures. These systems were oriented by the Orientations of Proteins in Membranes (OPM) database,^29^ and embedded in a lipid bilayer box, constructed using PACKMOL-memgen,^30^ containing 1-palmitoyl-2-oleoyl-*sn*-glycero-3-phosphocholine (POPC), cholesterol (CHL) (10:1 POPC:CHL ratio), water molecules (TIP3P), and monatomic Na^+^ and Cl^–^ ions (0.15 M). The resulting systems comprise between 225 and 250k atoms in a box of ∼120 Å × 120 Å × 140 Å (see the Supporting Information in the Zenodo repository for detailed values).

### 2.2 Molecular Dynamics Simulations

MD simulations of these models were performed with GROMACS2018.5.^31^ The amber14sb-ildn force field was used for the protein, solvent, and ions,^32^ a GROMACS adaptation of lipid14 for lipids,^33^ and the general Amber force field (GAFF2) with HF/6-31G*-derived RESP atomic charges for THC, THCV, and JWH-133.^34^ Molecular systems were subjected to 5000 steps of energy minimization, using the steepest descent algorithm, PME electrostatics, with the Verlet cutoff scheme. This was followed by a 25 ns equilibration protocol consisting of six steps, in which positional restraints are progressively removed, from all heavy atoms to only helix Cα carbons being restricted, meanwhile gradually reducing the applied forces, from 1000 kJ mol^–1^ nm^–2^ to 0 kJ mol^–1^ nm^–2^. After equilibration, three replicas of a 1 μs unrestrained MD trajectory were generated at a constant temperature of 300 K using separate v-rescale thermostats for the receptor, ligand, lipids, and solvent molecules. Initial velocities were randomly generated for each replica from a Maxwell distribution, using different random seeds. A time step of 2.0 fs was used for the integration of equations of motions using the leapfrog algorithm. Bonds involving hydrogen atoms were kept frozen by using the LINCS algorithm. Lennard-Jones interactions were computed using a cutoff of 1.1 nm under the Verlet cutoff scheme for neighbor searching, and the electrostatic interactions were treated using PME with the same real-space cutoff under periodic boundary conditions. Center of mass motion was removed from all systems. The Berendsen pressure control algorithm was chosen for equilibration and Parrinello–Rahman for production MDs. For complete details, see the Supporting Information in the Zenodo repository.

### 2.3 MD Analysis and Data Visualization

The analysis of the trajectories was performed using MDAnalysis;^35^ visualization and image rendering were performed with PyMOL^36^ and VMD,^37^ and graphical representations were obtained with the Seaborn Package.^38^

### 2.4 CB_1_R and CB_2_R Mutants

Mutations were produced using the QuikChange Site-Directed Mutagenesis Kit. The cDNA for hCB_1_R and hCB_2_R, cloned into pcDNA3.1, was amplified using sense and antisense primers harboring the triplets for the desired point mutation (Pfu turbo polymerase was used). The nonmutated DNA template was digested for 1 h with DpnI. PCR products were used to transform XL1-blue supercompetent cells. Finally, positive colonies were tested by sequencing to select those expressing the correct DNA sequence.

### 2.5 cAMP Determination Assays

Determination of cAMP levels in HEK-293T cells transiently expressing CB_1_R or CB_2_R (1 μg of cDNA) or the mutant version of the receptor was performed by using the Lance-Ultra cAMP kit (PerkinElmer). Two hours before initiating the experiment, the medium was substituted by a serum-free medium. Then, transfected cells were dispensed in white 384-well microplates at a density of 4000 cells per well and incubated for 15 min at room temperature with compounds, followed by 15 min incubation with forskolin, and 1 h more with homogeneous time-resolved fluorescence (HTRF) assay reagents. Fluorescence at 665 nm was analyzed on a PHERAstar Flagship microplate reader equipped with an HTRF optical module (BMG Labtech). Data analysis was made based on the fluorescence ratio emitted by the labeled cAMP probe (665 nm) over the light emitted by the europium cryptate-labeled anti-cAMP antibody (620 nm). A standard curve was used to calculate cAMP concentration. Forskolin-stimulated cAMP levels were normalized to 100%.

### 2.6 Pure Cannabinoids

Δ^9^-THC and Δ^9^-THCV substances were provided by Phytoplant Research S.L.U. Δ^9^-THC and Δ^9^-THCV were purified from the Moniek (CPVO/20160114) and Raquel (CPVO/20180114) varieties, respectively, using countercurrent chromatography as previously described.^39^ The purity of both cannabinoids was set at >95%.

## 3 Results

### 3.1 THC Adopts Two Distinct Binding Modes in CB_1_R But Not in CB_2_R

To understand the different molecular signatures of THC and THCV, at CB_1_R and CB_2_R, we first performed three replicate runs of unbiased 1 μs MD simulations of these compounds bound to the CB_1_R-G_i_ and CB_2_R-G_i_ complexes (see the Methods section). We have used G_i_-bound active states, instead of inactive structures, despite its higher computational cost, because agonists alone are not capable to stabilize the fully active conformation in the absence of the G protein, as shown by NMR experiments.^40^ Similarly, MD simulations of agonist bound to the inactive state of the receptor are not capable of reaching active-like conformations in the absence of the G protein. Moreover, MD simulations of active, G protein-bound, conformations have permitted to identify additional cavities to accommodate hydrophobic chains of ligands in sphingosine-1-phosphate^41^ and muscarinic^42^ receptors, which were not identified in similar simulations of inactive structures.

**Figure 2:**
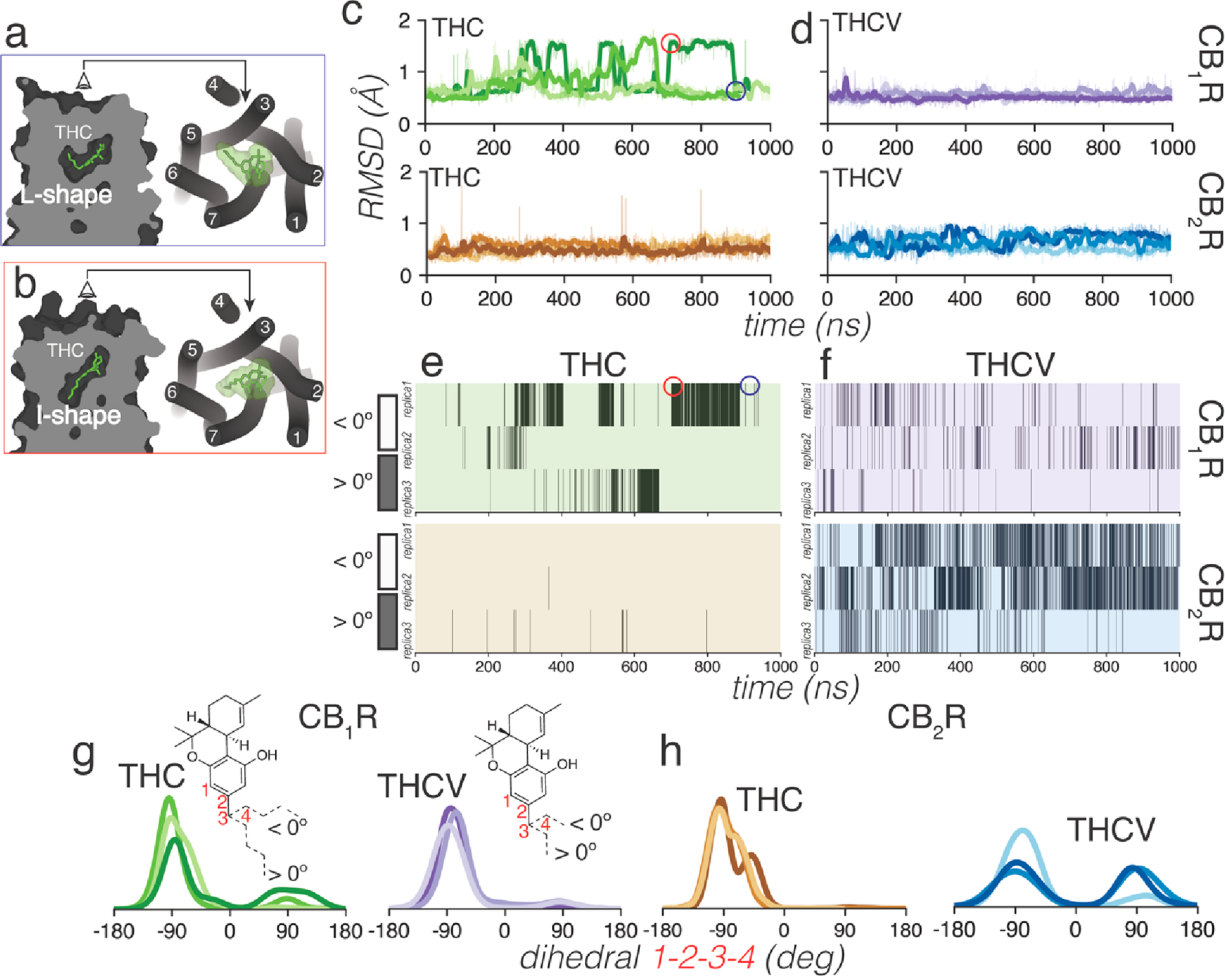
Conformational analysis of the alkyl chain of THC and THCV bound to CB_1_R and CB_2_R. Views parallel and perpendicular to the membrane plane of THC bound to CB_1_R in (a) an L-shape and (b) an I-shape conformation pointing toward cavities between either TMs 3 and 5 or TMs 3 and 6, respectively. Red and blue rectangles correspond to the structure in red and blue circles in panels (c) and (e). Rmsd values of ligand heavy atoms (c, d), heatmaps (e, f) showing the conformation of the alkyl chain (dihedral angle 1–2–3–4) in the *– anticlinal* (angle around −90° < 0°) or the *+ anticlinal* (around 90° > 0°), and histogram distribution of the dihedral angle 1–2–3–4 (g, h) during MD simulations of THC bound to CB_1_R (green lines or panels) or CB_2_R (brown) and THCV bound to CB_1_R (purple) or CB_2_R (blue). Three replicas of 1 μs of each complex were run.

THC and THCV were docked into these structures with the hydrophobic alkyl tail in the L-shape conformation, as observed in the cryo-EM structures of structurally similar ligands (see the Methods section). Root-mean-square deviations (rmsd) of the ligand heavy atoms show that THC visited during the MD simulations two different poses in the binding pocket of CB_1_R but not in CB_2_R (Figure 2c). In CB_1_R, THC adopts the initial L-shape conformation, in which the hydrophobic alkyl tail occupies a cavity between TMs 3 and 5 (Figure 2a), and an I-shape conformation, in which the alkyl tail occupies an intracellular cavity between TMs 3 and 6 (Figure 2b). The structure–function of the alkyl chain of THC has been reviewed,^43^ and this dual orientation is consistent with previous studies by others.^15,44^ The different conformation of the pentyl chain of THC is achieved by a change of a single dihedral angle in the chain, from *– anticlinal* (dihedral 1–2–3–4 around −90°) in the L-shape to *+ anticlinal* (around 90°) in the I-shape (Figure 2g). Heatmaps showing when THC adopts the *– anticlinal* (dihedral <0°) or *+ anticlinal* (>0°) conformation correlates with large (I-shape) and small (L-shape) rmsd values, respectively (Figures 2c and 2e). Notably, the pentyl chain of THC in CB_2_R rarely adopts the *+ anticlinal* conformation (Figure 2e), thus no large rmsd values could be observed from the initial L-shape conformation during the MD simulations (Figure 2c). The shorter 3-carbon propyl chain of THCV has fewer steric constraints and can visit the *+ anticlinal* conformation in both CB_1_R and CB_2_R simulations (Figures 2f–h).

**Figure 3:**
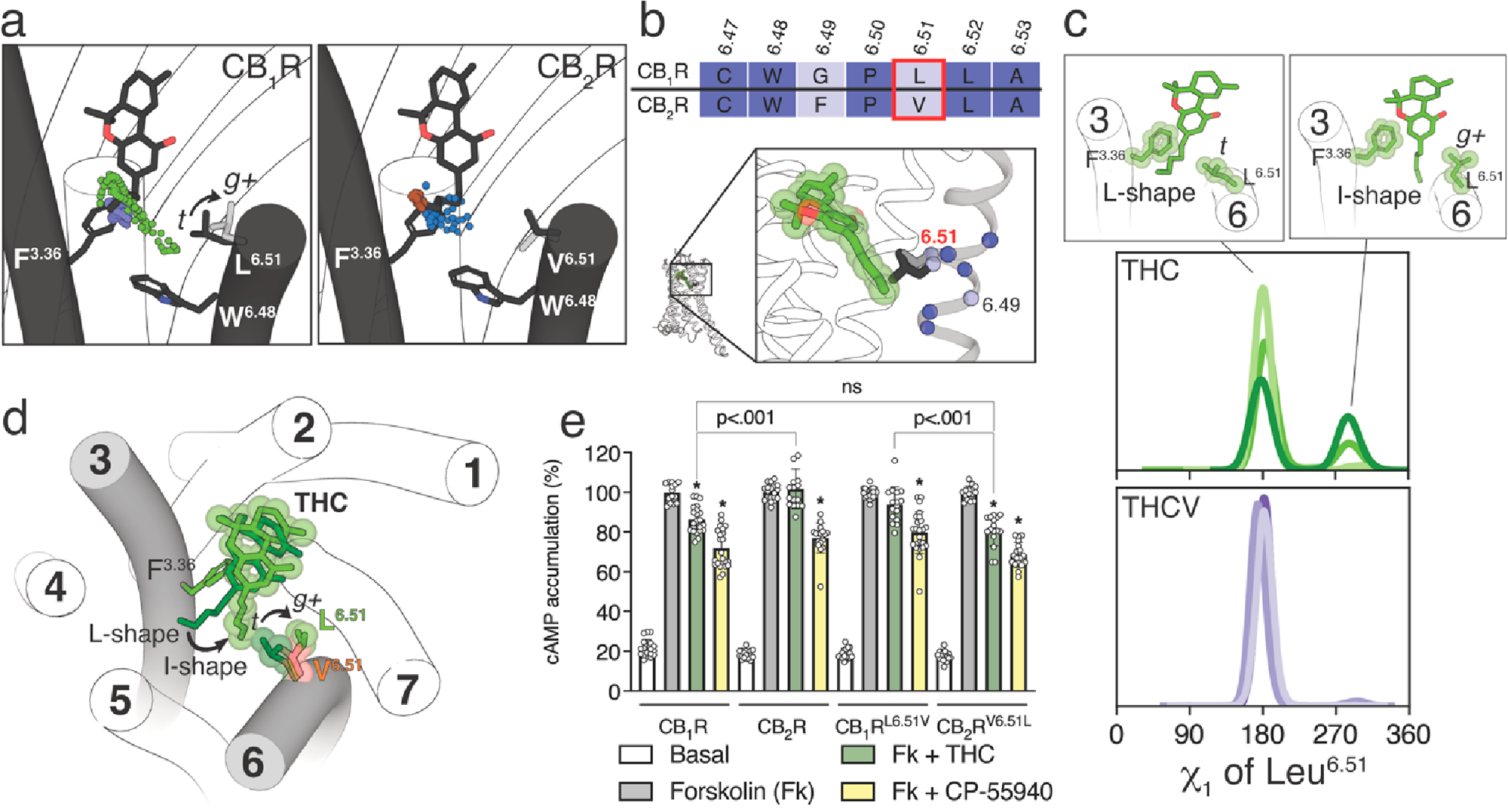
Conformational analysis of the Leu/Val^6.51^ side chains of CB_1_R and CB_2_R. (a) Evolution of the terminal methyl group (color spheres) of the alkyl chain of THC or THCV during MD simulations of THC bound to CB_1_R (green) or CB_2_R (brown) and THCV bound to CB_1_R (purple) or CB_2_R (blue). The black arrow represents the conformational change of the side chain of Leu^6.51^ from *t* to *g*+ that is triggered by the I-shaped conformation of THC in CB_1_R. (b) Sequence comparison of TM 6 between CB_1_R and CB_2_R and the position of these amino acids in CB_1_R (THC in the I-shaped conformation is shown as a reference). (c) Histogram distributions of the χ_1_ dihedral angle of Leu^6.51^ along the MD simulations of CB_1_R bound to THC or THCV. Leu^6.51^ adopted the *t* conformation with THCV and visited both the *t* and *g*+ conformations with THC. Representative structures of these conformations are also shown on the top panels. (d) Molecular representation of the different conformations of the five-carbon pentyl chain of THC (L- and I-shape) and Leu^6.51^ in CB_1_R (*t* and *g*+). The transition of the alkyl chain of THC from the L-shape to the I-shape conformation (black arrow), to fill the intracellular cavity between TMs 3 and 6 delineated by Phe^3.36^, Trp^6.48^, and Leu^6.51^, requires the conformational transition of Leu^6.51^ from *t* to *g*+ (black arrow). THC in the I-shape conformation and Leu^6.51^ are shown in VdW spheres to visualize the narrow size of the intracellular cavity. Val^6.51^ of CB_2_R (in orange) is superimposed to perceive that the β-branched character of the side chain blocks the access of the ligand chain to the intracellular cavity. (e) CAMP levels determined in HEK-293T cells transfected with CB_1_R, CB_2_R, CB_1_R^L6.51V^, or CB_2_R^V6.51L^. Cells were pretreated with vehicle (basal) or with THC (10 μM) or CP-55940 (100 nM) upon exposure to forskolin (Fk, 500 nM). Values are means ± SD (*n* = 4 with sixtiplicates in all experiments) of the percentage of forskolin-induced cAMP formation. These values were analyzed statistically with one-way ANOVA, followed by Bonferroni’s multiple comparison test (*: *p* < 0.05 compared with Fk).

It is not clear why the five-carbon pentyl chain of THC can also adopt the I-shape conformation, in which the alkyl tail occupies an intracellular cavity between TMs 3 and 6, in CB_1_R but not in CB_2_R. This intracellular cavity is delineated by the amino acid at position 6.51 (Figure 3a), which is Leu at CB_1_R and Val at CB_2_R (Figure 3b). The probability to undergo a side chain conformational change in Val is smaller than in Leu,^45^ due to the β-branched side chain that is shorter than the γ-branched side chain of Leu. Val is generally found with the γ-carbons flanking the small Hα in the *trans* (*t*, χ_1_ = 180°) rotamer conformation, whereas Leu can adopt the more stable *trans* (*t*, χ_1_ = 180°) and less stable *gauche+* (*g+*, χ_1_ = −60°) rotamer conformations, as observed in the dynameomics rotamer library.^46^Figure 3c shows the histogram distributions of the χ_1_ dihedral angle of Leu^6.51^ along the MD simulations of CB_1_R. These panels illustrate that the three-carbon propyl chain of THCV maintains Leu^6.51^ in the more stable *t* conformation during the simulation time, whereas the five-carbon pentyl chain of THC in the I-shape conformation triggers or stabilizes the *g+* conformation of Leu^6.51^ in CB_1_R, opening the access to the intracellular cavity between TMs 3 and 6 (Figure 3d). In contrast, the conformation of the bulky, β-branched, and more rigid side chain of Val^6.51^ in CB_2_R cannot be modified by the pentyl chain of THC (not shown), closing the access to the intracellular cavity.

### 3.2 The CB_1_R^L6.51V^ and CB_2_R^V6.51L^ Mutations Reverse the Pharmacology of THC

To experimentally validate the proposed different conformations of THC in CB_1_R and CB_2_R, we mutated Leu^6.51^ to Val in CB_1_R (CB_1_R^L6.51V^) and Val^6.51^ to Leu in CB_2_R (CB_2_R^V6.51L^), and we measured cAMP production in HEK-293T cells (Figure 3e). The nonselective CP-55940 agonist (100 nM) decreased, as expected for a G_i_-coupled receptor, cAMP formation induced by forskolin (500 nM), in a statistically significant manner, in wild-type CB_1_R and CB_2_R and mutant CB_1_R^L6.51V^ and CB_2_R^V6.51L^. In contrast, THC (10 μM) can significantly decrease forskolin-induced cAMP accumulation in CB_1_R but not in CB_2_R. We used high concentrations of THC to evaluate the greatest attainable response (ceiling effect). These results suggest that, in cAMP measurements, THC acts as a weak partial agonist only in CB_1_R. Remarkably, the pharmacological profile of THC changes in the mutant receptors. THC can significantly decrease forskolin-induced cAMP accumulation in CB_2_R^V6.51L^ but not in CB_1_R^L6.51V^. Moreover, cAMP accumulation induced by THC is not statistically different between CB_1_R and CB_2_R^V6.51L^. These experimental results, together with computational simulations, suggest that the residue at position 6.51, which is Leu at CB_1_R and Val at CB_2_R, is an additional element in the mechanism of receptor activation (see the Discussion section).

### 3.3 JWH-133 Activates CB_2_R via the Substituted Methyl Groups

**Figure 4:**
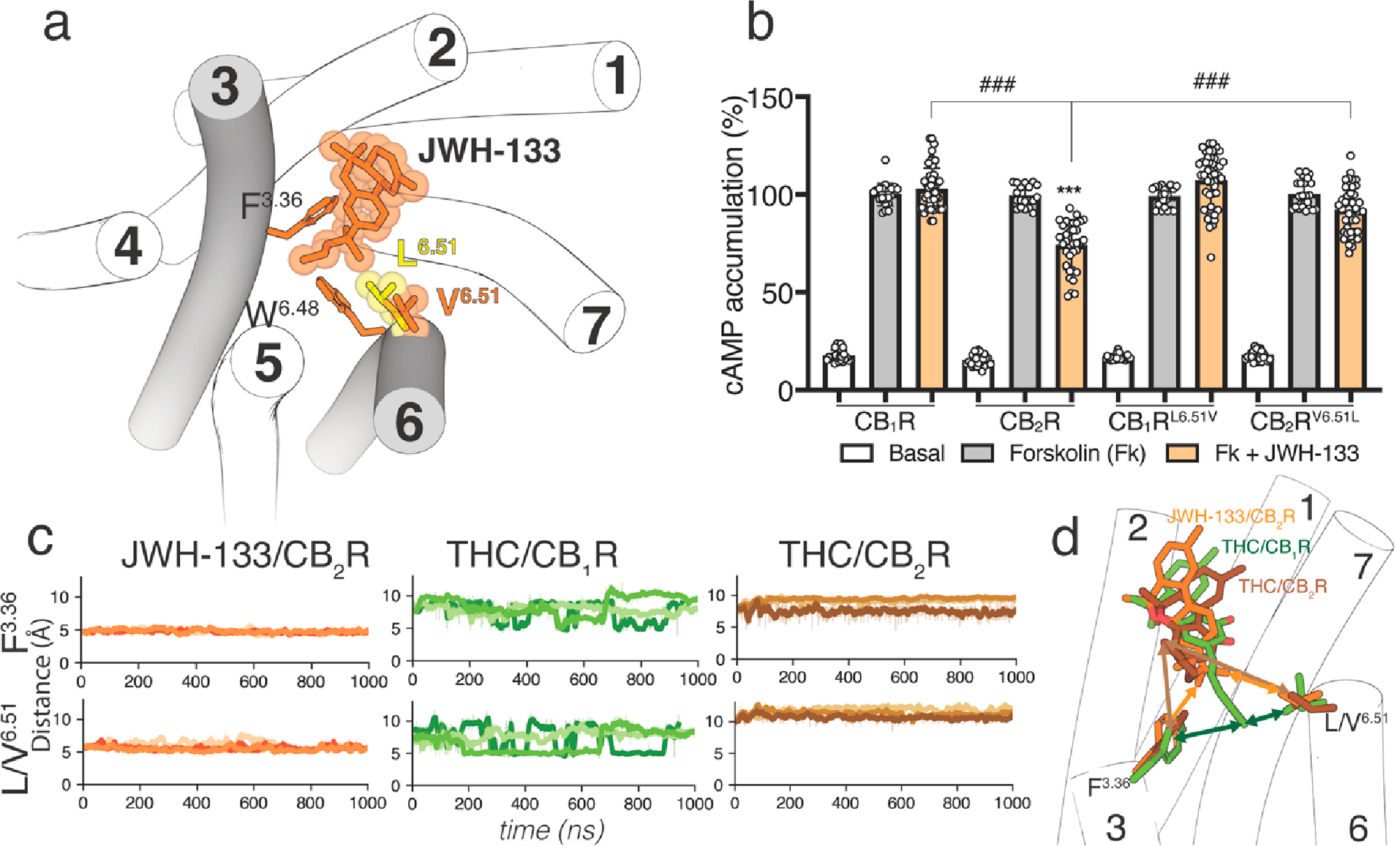
(a) The binding of JWH-133 to CB_2_R. Leu^6.51^ of CB_1_R (in yellow) is superimposed to perceive the longer side chain of Leu in *t* compared to Val. (b) cAMP levels determined in HEK-293T cells transfected with CB_1_R, CB_2_R, CB_1_R^L6.51V^, or CB_2_R^V6.51L^. Cells were pretreated with vehicle (basal) or with JWH-133 (100 nM) upon exposure to forskolin (Fk, 500 nM). Values are means ± SD (*n* = 4 with sixtiplicates in all experiments) of the percentage of forskolin-induced cAMP formation. These values were analyzed statistically with two-way ANOVA, followed by Bonferroni’s multiple comparison test ((***) *p* < 0.001 compared with Fk, (###) *p* < 0.001). (c) Distances between the terminal methyl group of the alkyl chain of THC and the dimethyl moiety of the dimethylbutyl chain of JWH-133 and the centroid of the aromatic ring of Phe^3.36^ and either the δ- or γ- carbon of Leu/Val^6.51^ of CB_1_R or CB_2_R (matching color arrows in panel (d) obtained during three replicas of MD simulations. (d) Comparison of the proposed binding modes of JWH-133 and THC in CB_2_R and THC in CB_1_R.

JWH-133 is a potent CB_2_R agonist, with little affinity for CB_1_R.^47^ The structure of JWH-133 is like THC and THCV with a 4-carbon butyl chain, instead of the 3-carbon propyl chain of THCV or the 5-carbon pentyl chain of THC (Figure 1). A significant difference between JWH-133 and either THC or THCV is the methyl substitutions on the chain (Figure 1). Branching close to the aromatic ring might restrict the dimethylbutyl chain conformation of JWH-133. Thus, it seems reasonable to study the molecular properties of JWH-133, as a full agonist, in complex with CB_2_R-G_i_ to challenge our proposed molecular models of THC and THCV (see section 3.1). Consequently, we performed simulations similar to those with THC and THCV to evaluate the binding mode of JWH-133 in CB_2_R (see the Methods section). The alkyl chain of JWH-133 always adopts the L-shape conformation in the *+ anticlinal* conformation, filling the cavity between TMs 3 and 5 (Figure 4a). The dimethyl moiety of the dimethylbutyl chain mediates hydrophobic interactions with Phe^3.36^ and Val^6.51^, during the simulation time (Figure 4c). To experimentally validate the key role of Val^6.51^ in JWH-133-induced CB_2_R activation, we measured the level of production of cAMP in CB_1_R^L6.51V^ and CB_2_R^V6.51L^ mutant receptors expressed in HEK-293T cells (Figure 4b). JWH-133 (100 nM) was unable to decrease forskolin-induced cAMP formation in CB_1_R but was statistically significantly lower in CB_2_R, as expected for a CB_2_R selective agonist. Substitution of Leu^6.51^ with Val in CB_1_R does not facilitate activation of CB_1_R^L6.51V^ by JWH-133. However, substitution of the single Val^6.51^ amino acid with Leu in CB_2_R makes JWH-133 unable to activate CB_2_R^V6.51L^. This points to both the bulky, β-branched, and rigid Val^6.51^ in CB_2_R and the bulky, branched dimethyl group of the dimethylbutyl chain of JWH-133 as key elements for CB_2_R activation (see the Discussion section). It was recently shown that the CB_2_R^V6.51L^ mutation also impeded HU308 and CP-55940, both containing the branched dimethyl group in the alkyl chain, to activate the G protein at CB_2_R.^48^

## 4 Discussion and Conclusions

**Figure 5:**
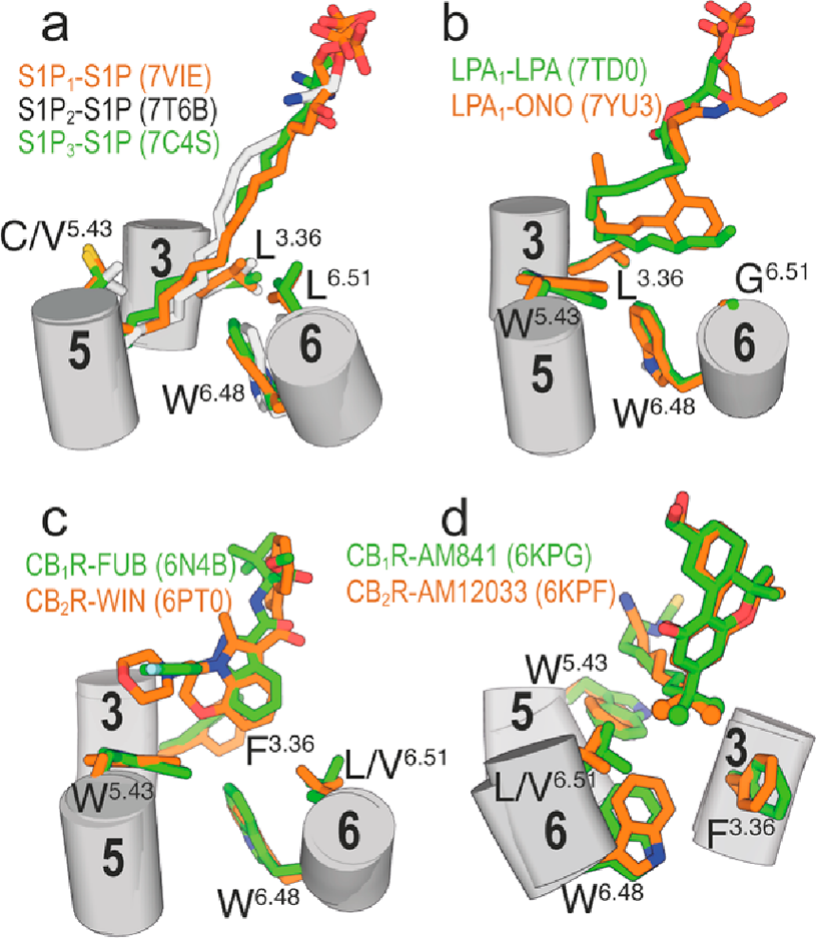
Cryo-EM structures of active S1P_1_, S1P_2_, or S1P_3_ bound to S1P (a); active LPA_1_ bound to LPA and ONO-0740556 (ONO) (b); active CB_1_R bound to MDMB-Fubinaca (FUB) (c) and AM841 (d); and active CB_2_R bound to WIN 55,212–2 (WIN) (c) and AM12033 (d). The branched dimethyl groups of AM841 and AM12033 are shown as spheres. Only key side chains at positions 3.36, 5.43, 6.48, and 6.51 are shown.

Among the ∼350 GPCRs for nonsensory functions, ∼35 are activated by hormone-like signaling molecules derived from lipid species with long hydrophobic chains.^6,49^ Some of these receptors possess distinctive structural signatures relative to other class A GPCRs such as the N-terminus and ECL-2 folding over the binding site,^50,51^ which causes the entry of the ligand to the orthosteric site through a tunnel formed between TMs 1 and 7;^52,53^ or lacking the highly conserved Pro^5.50^, part of the PIF motif that transmits the signal from the orthosteric ligand binding site to the G protein binding site.^54,55^ In PIF-containing GPCRs, the interaction of agonists with TM 5 triggers an inward movement of TM 5 at P^5.50^, a rotation of TM 3 at I^3.40^, and an outward movement of TM 6 at F^6.44^.^56,57^ In GPCRs lacking P^5.50^, agonists can alter the rotamer of the amino acid at position 3.36^20^ to trigger the rotation of TM 3 at I^3.40^ and outward movement of TM 6 at F^6.44^.^41^ For instance, in the active crystal structure of S1P_3_ bound to the endogenous agonist sphingosine-1-phosphate (S1P),^58^ the long hydrophobic side chain of d18:1 S1P binds in an extended conformation (I-shape) between TMs 4 and 5 (Figure 5a). S1P triggers conformational changes of Leu122^3.36^ from *t* to *g*+, among others (see quartet core in^58^), to accommodate the alkyl chain. Similar results were observed in S1P bound to S1P_1_^59^ and S1P_2_^60^ (Figure 5a). In the cryo-EM structure of active LPA_1_ bound to the lysophosphatidic acid (LPA),^61^ the alkyl chain of LPA cannot extend to the cleft between TMs 4 and 5 as S1P, due to the presence of the bulky Trp210^5.43^ in LPA_1_ (S1P_1–3_ possess the less bulky Cys206^5.43^, Val194^5.43^, or Cys200^5.43^), blocking the access. LPA adopts an U-shaped conformation bending backward and triggering the *g*+ conformation of Leu132^3.36^ (Figure 5b). The long acyl chain of the ONO-0740556 agonist, a more rigid and potent LPA analog, binds LPA_1_ in a different bent conformation than LPA^62^ (Figure 5c). In this case, the aromatic ring of ONO-0740556 triggers the *g*+ conformation of Leu132^3.36^. The lack of side chain in Gly274^6.51^ (LPA_1–3_ possess Gly at this key position) permits LPA_1_ to have a small pocket in front of Leu132^3.36^ encaging the terminus of LPA or the phenyl ring of ONO-0740556.^61,62^

CB_1_R and CB_2_R possess Trp279^5.43^ and Trp194^5.43^, respectively, thus blocking the TMs 4 and 5 cleft created in S1P binding to S1P_1–3_; and contain Phe200^3.36^ and Phe117^3.36^, respectively, as conformational toggle or trigger switch involved in the initial agonist-induced receptor activation. Notably, the indazole ring of MDMB-Fubinaca triggers the active *g*+ conformation of Phe200^3.36^ in CB_1_R by an aromatic–aromatic interaction (Figure 5c),^15^ and the aromatic core of WIN 55,212–2 also forms aromatic–aromatic interactions with Phe117^3.36^ in *g*+ of CB_2_R (Figure 5c).^17^ In other known structures of active CB_1_R and CB_2_R bound to agonists, the bulky and branched dimethyl groups of the alkyl chain of AM841 and AM12033 bridge Phe200^3.36^ in *g*+ and Leu359^6.51^ of CB_1_R and Phe117^3.36^ in *g*+ and Val261^6.51^ of CB_2_R (Figure 5d), respectively.^18^ The conformational change in Phe^3.36^, from pointing toward TM 6 in *t* to pointing toward TM 7 in *g*+, permits Trp^6.48^ to move toward TM 5 for receptor activation.

These data indicate that the hydrophobic alkyl chain of the signaling molecule is key in the process of ligand-induced receptor activation. Thus, in this paper, we have studied the conformation of the alkyl chain of THC, THCV, and JWH-133 bound to CB_1_R and CB_2_R and calculated their distances to Phe^3.36^ along the MD simulations. Among them, the distances between the terminal methyl group of the alkyl chain of THC and the centroid of the aromatic ring of Phe^3.36^ and either the δ- or γ- carbon of Leu/Val^6.51^ are important to highlight (see Figures 4c and 4d). They fluctuated from >5 Å to <5 Å in CB_1_R and are always >5 Å in CB_2_R, indicating a dual orientation of the alkyl chain in CB_1_R, which either occupies a cavity above Trp^5.43^ (between TMs 3 and 5) in an L-shape conformation, or an intracellular cavity between Phe^3.36^ and Trp^6.48^ (TMs 3 and 6) in an I-shape conformation (Figure 3d). The main achievement of this work is the discovery that THC in CB_1_R, but not in CB_2_R, can adopt this I-shape conformation. The intracellular cavity between Phe^3.36^ and Trp^6.48^ is also delineated by the amino acid at position 6.51, which is the γ-branched, flexible Leu side chain in CB_1_R and the β-branched, model rigid Val side chain in CB_2_R (Figure 3b). We have shown that the five-carbon pentyl chain of THC can trigger the conformational change of Leu^6.51^ from *t*, blocking the access of the chain to the intracellular cavity, to *g*+, opening the access (Figures 3c and 3d). This opening of the chain access to the intracellular cavity is not feasible with the rigid Val^6.51^ side chain of CB_2_R. The binding mode of THC in the I-shape conformation positions the alkyl chain between Phe^3.36^ in the active *g*+ conformation and Trp^6.48^ that is involved in the initial mechanism of agonist-induced receptor activation. Thus, these computational results are compatible with our experiments, showing that THC acts as a partial agonist in CB_1_R and as an antagonist in CB_2_R (Figure 3e).^12^ In agreement with our computational results, THC could not activate the mutant CB_1_R^L6.51V^ receptor and activated the mutant CB_2_R^V6.51L^ receptor as efficiently as wild type CB_1_R (Figure 3e). We have recently shown that the alkyl chain of cannabidiol, in the allosteric binding mode, also expands toward the intracellular cavity modulating the conformation of Phe^3.36^.^63^

The predicted binding mode of the dimethylbutyl chain conformation of the full agonist JWH-133 at CB_2_R is always in the L-shape conformation, filling the cavity between TMs 3 and 5 (Figure 4a). However, the branched dimethyl moiety of the ligand chain mediates hydrophobic interactions with Phe^3.36^ in the active *g*+ conformation and Val^6.51^ (Figure 4c). Notably, substitution of Val^6.51^ for Leu in CB_2_R makes JWH-133 unable to activate CB_2_R^V6.51L^ (Figure 4b). This supports the concept that the branched dimethylbutyl chain conformation of JWH-133 needs a foothold on the rigid Val^6.51^ to move Phe^3.36^ to the active *g*+ conformation for receptor activation.

In conclusion, our findings have shown that, in cannabinoid receptors and probably other receptors that recognize signaling molecules derived from lipid species with long hydrophobic chains, the amino acid at position 6.51 defines the size and shape of the cavity near Phe^3.36^ and Trp^6.48^ and is a key additional player in the mechanism of activation of this type of GPCRs.

## Data Availability

Input coordinates (.gro), topology files (.top), ligand parameters (.itp), input files, and representative structures collected during three replicas of MD simulations (in a PyMol session) of THC and THCV bound to CB_1_R and CB_2_R and JWH-133 bound to CB_2_R (the color code of the structures is as given in Figures 2–4) are available at https://zenodo.org/record/8114762. PACKMOL-Memgen, distributed with AmberTools, is free of charge; the Seaborn Package, MDAnalysis and GROMACS are open source; VMD is available to noncommercial users under a distribution-specific license; and PyMOL is commercial software with paid license.

## References

[ref1] Tahir M. N.; Shahbazi F.; Rondeau-Gagne S.; Trant J. F. The biosynthesis of the cannabinoids. J. Cannabis Res. 2021, 3 (1), 7 10.1186/s42238-021-00062-4. 33722296 PMC7962319

[ref2] Radwan M. M.; Chandra S.; Gul S.; ElSohly M. A. Cannabinoids, Phenolics, Terpenes and Alkaloids of Cannabis. Molecules 2021, 26 (9), 2774 10.3390/molecules26092774. 34066753 PMC8125862

[ref3] Jadoon K. A.; Ratcliffe S. H.; Barrett D. A.; Thomas E. L.; Stott C.; Bell J. D.; O’Sullivan S. E.; Tan G. D. Efficacy and Safety of Cannabidiol and Tetrahydrocannabivarin on Glycemic and Lipid Parameters in Patients With Type 2 Diabetes: A Randomized, Double-Blind, Placebo-Controlled, Parallel Group Pilot Study. Diabetes Care 2016, 39 (10), 1777–86. 10.2337/dc16-0650. 27573936

[ref4] Abioye A.; Ayodele O.; Marinkovic A.; Patidar R.; Akinwekomi A.; Sanyaolu A. Delta9-Tetrahydrocannabivarin (THCV): a commentary on potential therapeutic benefit for the management of obesity and diabetes. J. Cannabis Res. 2020, 2 (1), 6 10.1186/s42238-020-0016-7. 33526143 PMC7819335

[ref5] Cascio M. G.; Zamberletti E.; Marini P.; Parolaro D.; Pertwee R. G. The phytocannabinoid, Delta(9)-tetrahydrocannabivarin, can act through 5-HT(1)A receptors to produce antipsychotic effects. Br. J. Pharmacol. 2015, 172 (5), 1305–18. 10.1111/bph.13000. 25363799 PMC4337703

[ref6] Krishna Deepak R. N. V.; Verma R. K.; Hartono Y. D.; Yew W. S.; Fan H. Recent Advances in Structure, Function, and Pharmacology of Class A Lipid GPCRs: Opportunities and Challenges for Drug Discovery. Pharmaceuticals (Basel) 2022, 15 (1), 12 10.3390/ph15010012. PMC8779880 35056070

[ref7] Ferre S.; Ciruela F.; Dessauer C. W.; Gonzalez-Maeso J.; Hebert T. E.; Jockers R.; Logothetis D. E.; Pardo L. G protein-coupled receptor-effector macromolecular membrane assemblies (GEMMAs). Pharmacol. Ther. 2022, 231, 107977 10.1016/j.pharmthera.2021.107977. 34480967 PMC9375844

[ref8] Parsons L. H.; Hurd Y. L. Endocannabinoid signalling in reward and addiction. Nat. Rev. Neurosci. 2015, 16 (10), 579–94. 10.1038/nrn4004. 26373473 PMC4652927

[ref9] Morales P.; Hurst D. P.; Reggio P. H. Molecular Targets of the Phytocannabinoids: A Complex Picture. Prog. Chem. Org. Nat. Prod. 2017, 103, 103–131. 10.1007/978-3-319-45541-9_4. 28120232 PMC5345356

[ref10] Thomas A.; Stevenson L. A.; Wease K. N.; Price M. R.; Baillie G.; Ross R. A.; Pertwee R. G. Evidence that the plant cannabinoid Delta9-tetrahydrocannabivarin is a cannabinoid CB1 and CB2 receptor antagonist. Br. J. Pharmacol. 2005, 146 (7), 917–26. 10.1038/sj.bjp.0706414. 16205722 PMC1751228

[ref11] Zagzoog A.; Mohamed K. A.; Kim H. J. J.; Kim E. D.; Frank C. S.; Black T.; Jadhav P. D.; Holbrook L. A.; Laprairie R. B. In vitro and in vivo pharmacological activity of minor cannabinoids isolated from Cannabis sativa. Sci. Rep. 2020, 10 (1), 20405 10.1038/s41598-020-77175-y. 33230154 PMC7684313

[ref12] Raich I.; Rivas-Santisteban R.; Lillo A.; Lillo J.; Reyes-Resina I.; Nadal X.; Ferreiro-Vera C.; de Medina V. S.; Majellaro M.; Sotelo E.; Navarro G.; Franco R. Similarities and differences upon binding of naturally occurring Delta(9)-tetrahydrocannabinol-derivatives to cannabinoid CB1 and CB2 receptors. Pharmacol. Res. 2021, 174, 105970 10.1016/j.phrs.2021.105970. 34758399

[ref13] Shao Z.; Yin J.; Chapman K.; Grzemska M.; Clark L.; Wang J.; Rosenbaum D. M. High-resolution crystal structure of the human CB1 cannabinoid receptor. Nature 2016, 540 (7634), 602–606. 10.1038/nature20613. 27851727 PMC5433929

[ref14] Hua T.; Vemuri K.; Pu M.; Qu L.; Han G. W.; Wu Y.; Zhao S.; Shui W.; Li S.; Korde A.; Laprairie R. B.; Stahl E. L.; Ho J. H.; Zvonok N.; Zhou H.; Kufareva I.; Wu B.; Zhao Q.; Hanson M. A.; Bohn L. M.; Makriyannis A.; Stevens R. C.; Liu Z. J. Crystal Structure of the Human Cannabinoid Receptor CB1. Cell 2016, 167 (3), 750–762. 10.1016/j.cell.2016.10.004. 27768894 PMC5322940

[ref15] Krishna Kumar K.; Shalev-Benami M.; Robertson M. J.; Hu H.; Banister S. D.; Hollingsworth S. A.; Latorraca N. R.; Kato H. E.; Hilger D.; Maeda S.; Weis W. I.; Farrens D. L.; Dror R. O.; Malhotra S. V.; Kobilka B. K.; Skiniotis G. Structure of a Signaling Cannabinoid Receptor 1-G Protein Complex. Cell 2019, 176 (3), 448–458. 10.1016/j.cell.2018.11.040. 30639101 PMC6461403

[ref16] Li X.; Hua T.; Vemuri K.; Ho J. H.; Wu Y.; Wu L.; Popov P.; Benchama O.; Zvonok N.; Locke K.; Qu L.; Han G. W.; Iyer M. R.; Cinar R.; Coffey N. J.; Wang J.; Wu M.; Katritch V.; Zhao S.; Kunos G.; Bohn L. M.; Makriyannis A.; Stevens R. C.; Liu Z. J. Crystal Structure of the Human Cannabinoid Receptor CB2. Cell 2019, 176 (3), 459–467. 10.1016/j.cell.2018.12.011. 30639103 PMC6713262

[ref17] Xing C.; Zhuang Y.; Xu T. H.; Feng Z.; Zhou X. E.; Chen M.; Wang L.; Meng X.; Xue Y.; Wang J.; Liu H.; McGuire T. F.; Zhao G.; Melcher K.; Zhang C.; Xu H. E.; Xie X. Q. Cryo-EM Structure of the Human Cannabinoid Receptor CB2-Gi Signaling Complex. Cell 2020, 180 (4), 645–654. 10.1016/j.cell.2020.01.007. 32004460 PMC8247115

[ref18] Hua T.; Li X.; Wu L.; Iliopoulos-Tsoutsouvas C.; Wang Y.; Wu M.; Shen L.; Johnston C. A.; Nikas S. P.; Song F.; et al. Activation and Signaling Mechanism Revealed by Cannabinoid Receptor-Gi Complex Structures. Cell 2020, 180 (4), 655–665. 10.1016/j.cell.2020.01.008. 32004463 PMC7898353

[ref19] McAllister S. D.; Hurst D. P.; Barnett-Norris J.; Lynch D.; Reggio P. H.; Abood M. E. Structural mimicry in class A G protein-coupled receptor rotamer toggle switches: the importance of the F3.36(201)/W6.48(357) interaction in cannabinoid CB1 receptor activation. J. Biol. Chem. 2004, 279 (46), 48024–37. 10.1074/jbc.M406648200. 15326174

[ref20] Pellissier L. P.; Sallander J.; Campillo M.; Gaven F.; Queffeulou E.; Pillot M.; Dumuis A.; Claeysen S.; Bockaert J.; Pardo L. Conformational toggle switches implicated in basal constitutive and agonist-induced activated states of 5-hydroxytryptamine-4 receptors. Mol. Pharmacol. 2009, 75 (4), 982–990. 10.1124/mol.108.053686. 19168624

[ref21] Navarro G.; Gonzalez A.; Campanacci S.; Rivas-Santisteban R.; Reyes-Resina I.; Casajuana-Martin N.; Cordomi A.; Pardo L.; Franco R. Experimental and computational analysis of biased agonism on full-length and a C-terminally truncated adenosine A2A receptor. Comput. Struct. Biotechnol. J. 2020, 18, 2723–2732. 10.1016/j.csbj.2020.09.028. 33101610 PMC7550916

[ref22] Ersoy B. A.; Pardo L.; Zhang S.; Thompson D. A.; Millhauser G.; Govaerts C.; Vaisse C. Mechanism of N-terminal modulation of activity at the melanocortin-4 receptor GPCR. Nat. Chem. Biol. 2012, 8 (8), 725–30. 10.1038/nchembio.1008. 22729149 PMC3657613

[ref23] Maeda S.; Koehl A.; Matile H.; Hu H.; Hilger D.; Schertler G. F. X.; Manglik A.; Skiniotis G.; Dawson R. J. P.; Kobilka B. K. Development of an antibody fragment that stabilizes GPCR/G-protein complexes. Nat. Commun. 2018, 9 (1), 3712 10.1038/s41467-018-06002-w. 30213947 PMC6137068

[ref24] Webb B.; Sali A. Comparative Protein Structure Modeling Using MODELLER. Curr. Protoc. Bioinformatics 2016, 54, 5.6.1–5.6.32. 10.1002/cpbi.3. 25199792

[ref25] Marti-Renom M. A.; Stuart A. C.; Fiser A.; Sanchez R.; Melo F.; Sali A. Comparative protein structure modeling of genes and genomes. Annu. Rev. Biophys. Biomol. Struct. 2000, 29, 291–325. 10.1146/annurev.biophys.29.1.291. 10940251

[ref26] Dolinsky T. J.; Nielsen J. E.; McCammon J. A.; Baker N. A. PDB2PQR: an automated pipeline for the setup of Poisson-Boltzmann electrostatics calculations. Nucleic Acids Res. 2004, 32 (Web Server issue), W665–W667. 10.1093/nar/gkh381. 15215472 PMC441519

[ref27] Sondergaard C. R.; Olsson M. H.; Rostkowski M.; Jensen J. H. Improved Treatment of Ligands and Coupling Effects in Empirical Calculation and Rationalization of pKa Values. J. Chem. Theory Comput. 2011, 7 (7), 2284–95. 10.1021/ct200133y. 26606496

[ref28] Mayol E.; Garcia-Recio A.; Tiemann J. K. S.; Hildebrand P. W.; Guixa-Gonzalez R.; Olivella M.; Cordomi A. HomolWat: a web server tool to incorporate ‘homologous’ water molecules into GPCR structures. Nucleic Acids Res. 2020, 48 (W1), W54–W59. 10.1093/nar/gkaa440. 32484557 PMC7319549

[ref29] Lomize M. A.; Pogozheva I. D.; Joo H.; Mosberg H. I.; Lomize A. L. OPM database and PPM web server: resources for positioning of proteins in membranes. Nucleic Acids Res. 2012, 40 (D1), D370–D376. 10.1093/nar/gkr703. 21890895 PMC3245162

[ref30] Schott-Verdugo S.; Gohlke H. PACKMOL-Memgen: A Simple-To-Use, Generalized Workflow for Membrane-Protein-Lipid-Bilayer System Building. J. Chem. Inf. Model. 2019, 59 (6), 2522–2528. 10.1021/acs.jcim.9b00269. 31120747

[ref31] Abraham M. J.; Murtola T.; Schulz R.; Pall S.; Smith J. C.; Hess B.; Lindahl E. GROMACS: High performance molecular simulations through multi-level parallelism from laptops to supercomputers. SoftwareX 2015, 1–2, 19–25. 10.1016/j.softx.2015.06.001.

[ref32] Maier J. A.; Martinez C.; Kasavajhala K.; Wickstrom L.; Hauser K. E.; Simmerling C. ff14SB: Improving the Accuracy of Protein Side Chain and Backbone Parameters from ff99SB. J. Chem. Theory Comput. 2015, 11 (8), 3696–713. 10.1021/acs.jctc.5b00255. 26574453 PMC4821407

[ref33] Dickson C. J.; Madej B. D.; Skjevik A. A.; Betz R. M.; Teigen K.; Gould I. R.; Walker R. C. Lipid14: The Amber Lipid Force Field. J. Chem. Theory Comput. 2014, 10 (2), 865–879. 10.1021/ct4010307. 24803855 PMC3985482

[ref34] Wang J.; Wolf R. M.; Caldwell J. W.; Kollman P. A.; Case D. A. Development and testing of a general amber force field. J. Comput. Chem. 2004, 25 (9), 1157–74. 10.1002/jcc.20035. 15116359

[ref35] Michaud-Agrawal N.; Denning E. J.; Woolf T. B.; Beckstein O. MDAnalysis: a toolkit for the analysis of molecular dynamics simulations. J. Comput. Chem. 2011, 32 (10), 2319–27. 10.1002/jcc.21787. 21500218 PMC3144279

[ref36] The PyMOL Molecular Graphics System, Version 1.3r1; Schrodinger, LLC, 2010.

[ref37] Humphrey W.; Dalke A.; Schulten K. VMD: visual molecular dynamics. J. Mol. Graph. 1996, 14 (1), 33–8. 10.1016/0263-7855(96)00018-5. 8744570

[ref38] Waskom M. L. seaborn: statistical data visualization. J. Open Source Softw. 2021, 6 (60), 3021 10.21105/joss.03021.

[ref39] Nadal X. Methods of purifying cannabinoids using liquid:liquid chromatography. US10207199B2, 2019.

[ref40] Nygaard R.; Zou Y.; Dror R. O.; Mildorf T. J.; Arlow D. H.; Manglik A.; Pan A. C.; Liu C. W.; Fung J. J.; Bokoch M. P.; Thian F. S.; Kobilka T. S.; Shaw D. E.; Mueller L.; Prosser R. S.; Kobilka B. K. The Dynamic Process of beta(2)-Adrenergic Receptor Activation. Cell 2013, 152 (3), 532–42. 10.1016/j.cell.2013.01.008. 23374348 PMC3586676

[ref41] Troupiotis-Tsailaki A.; Zachmann J.; Gonzalez-Gil I.; Gonzalez A.; Ortega-Gutierrez S.; Lopez-Rodriguez M. L.; Pardo L.; Govaerts C. Ligand chain length drives activation of lipid G protein-coupled receptors. Sci. Rep. 2017, 7 (1), 2020 10.1038/s41598-017-02104-5. 28515494 PMC5435731

[ref42] Powers A. S.; Pham V.; Burger W. A. C.; Thompson G.; Laloudakis Y.; Barnes N. W.; Sexton P. M.; Paul S. M.; Christopoulos A.; Thal D. M.; Felder C. C.; Valant C.; Dror R. O. Structural basis of efficacy-driven ligand selectivity at GPCRs. Nat. Chem. Biol. 2023, 19, 805 10.1038/s41589-022-01247-5. 36782010 PMC10299909

[ref43] Bow E. W.; Rimoldi J. M. The Structure-Function Relationships of Classical Cannabinoids: CB1/CB2Modulation. Perspect. Medicin. Chem. 2016, 8, 17–39. 10.4137/PMC.S32171. 27398024 PMC4927043

[ref44] Jung S. W.; Cho A. E.; Yu W. Exploring the Ligand Efficacy of Cannabinoid Receptor 1 (CB1) using Molecular Dynamics Simulations. Sci. Rep. 2018, 8 (1), 13787 10.1038/s41598-018-31749-z. 30213978 PMC6137198

[ref45] Gaudreault F.; Chartier M.; Najmanovich R. Side-chain rotamer changes upon ligand binding: common, crucial, correlate with entropy and rearrange hydrogen bonding. Bioinformatics 2012, 28 (18), i423–i430. 10.1093/bioinformatics/bts395. 22962462 PMC3436822

[ref46] Scouras A. D.; Daggett V. The Dynameomics rotamer library: amino acid side chain conformations and dynamics from comprehensive molecular dynamics simulations in water. Protein Sci. 2011, 20 (2), 341–52. 10.1002/pro.565. 21280126 PMC3048419

[ref47] Huffman J. W.; Liddle J.; Yu S.; Aung M. M.; Abood M. E.; Wiley J. L.; Martin B. R. 3-(1′,1′-Dimethylbutyl)-1-deoxy-delta8-THC and related compounds: synthesis of selective ligands for the CB2 receptor. Bioorg. Med. Chem. Lett. 1999, 7 (12), 2905–14. 10.1016/S0968-0896(99)00219-9. 10658595

[ref48] Li X.; Chang H.; Bouma J.; de Paus L. V.; Mukhopadhyay P.; Paloczi J.; Mustafa M.; van der Horst C.; Kumar S. S.; Wu L.; Yu Y.; van den Berg R.; Janssen A. P. A.; Lichtman A.; Liu Z. J.; Pacher P.; van der Stelt M.; Heitman L. H.; Hua T. Structural basis of selective cannabinoid CB(2) receptor activation. Nat. Commun. 2023, 14 (1), 1447 10.1038/s41467-023-37112-9. 36922494 PMC10017709

[ref49] Alexander S. P.; Christopoulos A.; Davenport A. P.; Kelly E.; Marrion N. V.; Peters J. A.; Faccenda E.; Harding S. D.; Pawson A. J.; Sharman J. L.; Southan C.; Davies J. A. the Concise Guide to Pharmacology 2017/18: G protein-coupled receptors. Br. J. Pharmacol. 2017, 174 (Suppl 1), S17–S129. 10.1111/bph.13878. 29055040 PMC5650667

[ref50] Gonzalez A.; Cordomí A.; Caltabiano G.; Pardo L. Impact of helix irregularities on sequence alignment and homology modelling of G protein-coupled receptors. Chembiochem 2012, 13 (10), 1393–1399. 10.1002/cbic.201200189. 22761034

[ref51] Audet M.; Stevens R. C. Emerging structural biology of lipid G protein-coupled receptors. Protein Sci. 2019, 28 (2), 292–304. 10.1002/pro.3509. 30239054 PMC6319753

[ref52] Stanley N.; Pardo L.; Fabritiis G. D. The pathway of ligand entry from the membrane bilayer to a lipid G protein-coupled receptor. Sci. Rep. 2016, 6, 22639 10.1038/srep22639. 26940769 PMC4778059

[ref53] Casajuana-Martin N.; Navarro G.; Gonzalez A.; Llinas Del Torrent C.; Gomez-Autet M.; Quintana Garcia A.; Franco R.; Pardo L. A Single Point Mutation Blocks the Entrance of Ligands to the Cannabinoid CB(2) Receptor via the Lipid Bilayer. J. Chem. Inf. Model. 2022, 62 (22), 5771–5779. 10.1021/acs.jcim.2c00865. 36302505 PMC9709915

[ref54] Weis W. I.; Kobilka B. K. The Molecular Basis of G Protein-Coupled Receptor Activation. Annu. Rev. Biochem. 2018, 87, 897–919. 10.1146/annurev-biochem-060614-033910. 29925258 PMC6535337

[ref55] Zhou Q.; Yang D.; Wu M.; Guo Y.; Guo W.; Zhong L.; Cai X.; Dai A.; Jang W.; Shakhnovich E. I. Common activation mechanism of class A GPCRs. Elife 2019, 8, 10.7554/eLife.50279. PMC6954041 31855179

[ref56] Sansuk K.; Deupi X.; Torrecillas I. R.; Jongejan A.; Nijmeijer S.; Bakker R. A.; Pardo L.; Leurs R. A Structural Insight into the Reorientation of Transmembrane Domains 3 and 5 during Family A G Protein-Coupled Receptor Activation. Mol. Pharmacol. 2011, 79 (2), 262–9. 10.1124/mol.110.066068. 21081645

[ref57] Rasmussen S. G.; Choi H. J.; Fung J. J.; Pardon E.; Casarosa P.; Chae P. S.; Devree B. T.; Rosenbaum D. M.; Thian F. S.; Kobilka T. S.; Schnapp A.; Konetzki I.; Sunahara R. K.; Gellman S. H.; Pautsch A.; Steyaert J.; Weis W. I.; Kobilka B. K. Structure of a nanobody-stabilized active state of the beta(2) adrenoceptor. Nature 2011, 469 (7329), 175–80. 10.1038/nature09648. 21228869 PMC3058308

[ref58] Maeda S.; Shiimura Y.; Asada H.; Hirata K.; Luo F.; Nango E.; Tanaka N.; Toyomoto M.; Inoue A.; Aoki J.; Iwata S.; Hagiwara M. Endogenous agonist-bound S1PR3 structure reveals determinants of G protein-subtype bias. Sci. Adv. 2021, 7 ( (24), ), 10.1126/sciadv.abf5325. PMC8189593 34108205

[ref59] Yu L.; He L.; Gan B.; Ti R.; Xiao Q.; Yang X.; Hu H.; Zhu L.; Wang S.; Ren R. Structural insights into sphingosine-1-phosphate receptor activation. Proc. Natl. Acad. Sci. U.S.A. 2022, 119 (16), e2117716119 10.1073/pnas.2117716119. 35412894 PMC9169846

[ref60] Chen H.; Chen K.; Huang W.; Staudt L. M.; Cyster J. G.; Li X. Structure of S1PR2-heterotrimeric G(13) signaling complex. Sci. Adv. 2022, 8 (13), eabn0067 10.1126/sciadv.abn0067. 35353559 PMC8967229

[ref61] Liu S.; Paknejad N.; Zhu L.; Kihara Y.; Ray M.; Chun J.; Liu W.; Hite R. K.; Huang X. Y. Differential activation mechanisms of lipid GPCRs by lysophosphatidic acid and sphingosine 1-phosphate. Nat. Commun. 2022, 13 (1), 731 10.1038/s41467-022-28417-2. 35136060 PMC8826421

[ref62] Akasaka H.; Tanaka T.; Sano F. K.; Matsuzaki Y.; Shihoya W.; Nureki O. Structure of the active G(i)-coupled human lysophosphatidic acid receptor 1 complexed with a potent agonist. Nat. Commun. 2022, 13 (1), 5417 10.1038/s41467-022-33121-2. 36109516 PMC9477835

[ref63] Navarro G.; Gonzalez A.; Sanchez-Morales A.; Casajuana-Martin N.; Gomez-Ventura M.; Cordomi A.; Busque F.; Alibes R.; Pardo L.; Franco R. Design of Negative and Positive Allosteric Modulators of the Cannabinoid CB2 Receptor Derived from the Natural Product Cannabidiol. J. Med. Chem. 2021, 64 (13), 9354–9364. 10.1021/acs.jmedchem.1c00561. 34161090

